# MicroRNA-101 Regulates 6-Hydroxydopamine-Induced Cell Death by Targeting Suppressor/Enhancer Lin-12-Like in SH-SY5Y Cells

**DOI:** 10.3389/fnmol.2021.748026

**Published:** 2021-12-09

**Authors:** Tomohiro Omura, Luna Nomura, Ran Watanabe, Hiroki Nishiguchi, Kazuhiro Yamamoto, Satoshi Imai, Shunsaku Nakagawa, Kotaro Itohara, Atsushi Yonezawa, Takayuki Nakagawa, Junichi Kunimasa, Ikuko Yano, Kazuo Matsubara

**Affiliations:** ^1^Department of Pharmacy, Kobe University Hospital, Kobe, Japan; ^2^Department of Clinical Pharmacology and Therapeutics, Kyoto University Hospital, Kyoto, Japan; ^3^Education and Research Center for Clinical Pharmacy, Kobe Pharmaceutical University, Kobe, Japan; ^4^Graduate School of Pharmaceutical Sciences, Kyoto University, Kyoto, Japan; ^5^Department of Pharmacy, Wakayama Medical University Hospital, Wakayama, Japan

**Keywords:** miR-101, Parkinson’s disease, HMG-CoA reductase degradation 1 (HRD1), suppressor/enhancer lin-12-like (SEL1L), microRNA, endoplasmic reticulum stress (ER stress)

## Abstract

Endoplasmic reticulum (ER) stress has been reported as a cause of Parkinson’s disease (PD). We have previously reported that the ubiquitin ligase HMG-CoA reductase degradation 1 (HRD1) and its stabilizing factor suppressor/enhancer lin-12-like (SEL1L) participate in the ER stress. In addition, we recently demonstrated that neuronal cell death is enhanced in the cellular PD model when SEL1L expression is suppressed compared with cell death when HRD1 expression is suppressed. This finding suggests that SEL1L is a critical key molecule in the strategy for PD therapy. Thus, investigation into whether microRNAs (miRNAs) regulate SEL1L expression in neurons should be interesting because relationships between miRNAs and the development of neurological diseases such as PD have been reported in recent years. In this study, using miRNA databases and previous reports, we searched for miRNAs that could regulate SEL1L expression and examined the effects of this regulation on cell death in PD models created by 6-hydroxydopamine (6-OHDA). Five miRNAs were identified as candidate miRNAs that could modulate SEL1L expression. Next, SH-SY5Y cells were exposed to 6-OHDA, following which miR-101 expression was found to be inversely correlated with SEL1L expression. Therefore, we selected miR-101 as a candidate miRNA for SEL1L modulation. We confirmed that miR-101 directly targets the SEL1L 3′ untranslated region, and an miR-101 mimic suppressed the 6-OHDA–induced increase in SEL1L expression and enhanced cell death. Furthermore, an miR-101 inhibitor suppressed this response. These results suggest that miR-101 regulates SEL1L expression and may serve as a new target for PD therapy.

## Introduction

Parkinson’s disease (PD), the second most prevalent neurodegenerative disorder after Alzheimer’s disease, is characterized by movement disorders (Kalia and Lang, 2015). In patients with PD, the dopamine content in the striatum is decreased because of the loss of dopaminergic neurons in the substantia nigra pars compacta in the midbrain (Damier et al., 1999). PD pathogenesis is considered to be associated with mitochondrial dysfunction, oxidative stress, and endoplasmic reticulum (ER) stress caused by aging combined with genetic and/or environmental factors (Matsubara et al., 1998; Mattson, 2000; Shimizu et al., 2003; Stefani et al., 2012).

Endoplasmic reticulum has important functions, including protein synthesis, folding, and glycosylation. Disruption of these functions by various stressors leads to the accumulation of unfolded proteins in the ER, resulting in cell death; this process is termed ER stress. In eukaryotic cells, the ER stress activates a physiological response called the unfolded protein response (UPR) in order to avoid the accumulation of unfolded proteins (Schroder and Kaufman, 2005). In ER-associated degradation (ERAD), a mechanism of the UPR system, accumulated proteins are retro-translocated from the ER to the cytosol through the translocon and polyubiquitinated by ubiquitin-conjugating enzymes, E3 ubiquitin ligase, and other components. Finally, these polyubiquitinated unfolded proteins are degraded by the 26S proteasome (Hershko and Ciechanover, 1998; Yoshida, 2007).

The ubiquitin ligase HMG-CoA reductase degradation 1 (HRD1) is involved in the ER stress, and suppressor/enhancer lin-12-like (SEL1L) has been identified as an HRD1 stabilizer (Kaneko et al., 2002; Iida et al., 2011). During the ER stress, mammalian cells suppress cell death by triggering HRD1/SEL1L-mediated protein degradation (Iida et al., 2011; Sun et al., 2014). We previously found that HRD1 was localized in dopaminergic neurons in the substantia nigra pars compacta of the midbrain (Omura et al., 2008). Additionally, we found that HRD1 interacted with and degraded parkin-associated endothelin receptor-like receptor (Pael-R), a substrate of the ubiquitin ligase parkin (Imai et al., 2001), resulting in the suppression of Pael-R–induced cell death (Omura et al., 2006). In patients with autosomal recessive juvenile PD, unfolded Pael-R accumulates in the ER, causing ER stress-induced cell death because of the loss of parkin function induced by genetic mutations (Imai et al., 2001). We also demonstrated that HRD1 alleviated neuronal cell death in a cellular model of PD using 6-hydroxydopamine (6-OHDA), which is widely used in *in vitro* and *in vivo* models of PD (Hernandez-Baltazar et al., 2017; Salari and Bagheri, 2019). Conversely, cell death was enhanced even when SEL1L expression was suppressed in a PD model (Omura et al., 2018), suggesting an important role for SEL1L in the pathogenesis of PD.

MicroRNAs (miRNAs) are short non-coding RNAs, 20–25 bases in length, that play critical roles in many biological processes, including cellular proliferation, differentiation, maturation, immune homeostasis, and normal cellular function (Schickel et al., 2008). MiRNAs negatively regulate gene expression through mRNA degradation or post-transcriptional inhibition by interacting with the 3′-untranslated region (UTR) of target mRNAs, thereby influencing the expression of various proteins (Filipowicz et al., 2008). Previously published studies have demonstrated that miRNAs are involved in the development of various diseases, including PD (Ardekani and Naeini, 2010; Chen et al., 2016).

MicroRNAs that regulate SEL1L expression have been observed in human pancreatic ductal adenocarcinoma and murine neural stem cells (Cardano et al., 2011; Liu et al., 2014) but not in human neurons. Therefore, we searched for miRNAs that regulated SEL1L expression in human neurons and examined whether the identified miRNAs affected cell viability in PD.

## Materials and Methods

### Search for MicroRNAs Targeting Suppressor/Enhancer Lin-12-Like in Four Databases

Using four public databases, i.e., Targetscan, PicTar, miRanda, and miRDB, potential miRNAs targeting SEL1L were searched. Among the identified miRNAs from each database, those that were common across all databases were selected as candidate miRNAs.

### Chemical Reagents and Antibodies

6-Hydroxydopamine hydrobromide was purchased from Sigma-Aldrich (St. Louis, MO, United States) and 3-(4,5-dimethyl-2-thiazolyl)-2, 5-diphenyl-2H-tetrazolium bromide (MTT) was obtained from Dojindo Laboratories (Kumamoto, Japan). 0.4% trypan blue solution was purchased from Wako Pure Chemicals (Osaka, Japan). All chemicals (Wako Pure Chemicals) used in the experiments belonged either to the highest or analytical grade. An antibody targeting β-actin (A1978) was purchased from Sigma-Aldrich. Anti-SEL1L (sc377350) and anti-HRD1/SYVN1 (Ab170901) antibodies were obtained from Santa Cruz Biotechnology (Santa Cruz, CA, United States) and Abcam (Cambridge, United Kingdom), respectively. Horseradish peroxidase-conjugated goat anti-rabbit (cat. no. 7074S) and anti-mouse (cat. no. 7076S) secondary antibodies were purchased from Cell Signaling Technology (Danvers, MA, United States).

### Cell Culture and Drug Treatment

SH-SY5Y human neuroblastoma cells were cultured in Dulbecco’s modified Eagle’s medium supplemented with 10% fetal bovine serum (Thermo Fisher Scientific, Waltham, MA, United States) in a humidified atmosphere with 5% CO_2_ at 37°C. Drugs were added to cells seeded in 24-well plates for the MTT, trypan blue exclusion (TBE), and luciferase assays and in 6-well plates for real-time polymerase chain reaction (PCR) and western blotting.

### MTT Assay

Cells were incubated with 50 μl of 5 mg/ml MTT (final concentration, 0.5 mg/ml) for 30 min at 37°C. After discarding the medium, stained cells were dissolved in 1 ml dimethyl sulfoxide. Finally, the optical density was measured at 560 nm (reference wavelength, 630 nm) with a microplate reader (VERSAmax, Molecular Devices, Sun Jose, CA, United States).

### Trypan Blue Exclusion Assay

Cells were washed with phosphate-buffered saline (PBS) buffer, and trypsin was added to each well. The cell suspensions were centrifuged at 200 × *g* for 5 min, and the supernatants were removed. The cell pellets were resuspended in 100 μl PBS buffer and 70 μl of the cell suspension was stained with 70 μl of 0.4% trypan blue solution. Cell counts and viability were visually determined using a hemocytometer and a light microscope.

### RNA Isolation and Quantitative Real-Time Polymerase Chain Reaction for mRNA and MicroRNA

Total RNA was isolated from SH-SY5Y cells using a mirVana™ miRNA Isolation Kit (Ambion, Austin, TX, United States) according to the manufacturer’s instructions. For miRNA quantitation, cDNA was synthesized from RNA (15 ng) with specific miRNA reverse transcriptase primers (Thermo Fisher Scientific) using a TaqMan MicroRNA Reverse Transcription kit (Thermo Fisher Scientific). The mRNA levels were quantitated as follows: cDNA was generated from RNA (1 μg) using a High Capacity RNA-to-cDNA Kit (Thermo Fisher Scientific). Quantitative real-time PCR was performed according to the instructions of the StepOnePlus Real-Time PCR System (Thermo Fisher Scientific) with TaqMan Fast Advanced Master Mix (Thermo Fisher Scientific) for mRNA quantitation and a TaqMan MicroRNA Assay kit (Thermo Fisher Scientific) for miRNA quantitation. Probe–primer solutions for mRNA (obtained from Thermo Fisher Scientific) specific for HRD1 (Hs00381211_m1), SEL1L (Hs01071406_m1), and 18S rRNA (Hs99999901_s1) were used. Probes for miRNAs (purchased from Thermo Fisher Scientific) specific for the hsa-miR-155 (assay ID 002623), hsa-miR-183 (assay ID 002269), hsa-miR-101 (assay ID 002253), hsa-miR-181a-2 (assay ID 002317), hsa-miR-181b (assay ID 462578_mat) and RNU44 (assay ID 001094) genes were used. 18S rRNA and RNU44 were used as internal controls to normalize mRNA and miRNA expression, respectively.

### Luciferase Reporter Assay

Expression vectors containing the 3′-UTR fragments of SEL1L were purchased from GeneCopoeia (Rockville, MD, United States). As the 3′-UTR of SEL1L is >4,000 bp in length, it was divided into shorter fragments and two constructs with overlapping sequences were delivered. Then, the SEL1L 3′-UTR cDNA fragment (approximately 500 bp) containing the miR-101 binding site was amplified and subcloned into a pmirGLO plasmid (Promega, Madison, WI, United States) from Kazusa Genome Technologies (Chiba, Japan). We examined this entire 3′-UTR of SEL1L and confirmed that the binding site for miR-101 was present only in the abovementioned 500-bp region. The mutant 3′-UTR, which contained the mutated sequence of the complementary site for the seed region of miR-101, was generated by Kazusa Genome Technologies. For the luciferase reporter assay, SH-SY5Y cells were cotransfected with a miR-101 mimic (assay ID MC11414, Thermo Fisher Scientific), a miRNA mimic negative control #1 (Thermo Fisher Scientific), and luciferase reporter constructs containing the wild-type SEL1L 3′-UTR (pmirGLO-SEL1L-3′-UTR-WT) or mutant SEL1L 3′-UTR (pmirGLO-SEL1L-3′-UTR-MUT) using Lipofectamine 2000 (Thermo Fisher Scientific). Luciferase activity was measured using the Dual-Glo Reporter Assay System (Promega). Firefly luciferase activity was normalized to *Renilla* luciferase activity in each transfected well.

### MicroRNA Mimic or Inhibitor Transfection

The specific miR-101 mimic, miR-101 inhibitor (assay ID MH11414), miRNA mimic negative control #1, and miRNA inhibitor negative control #1 were purchased from Thermo Fisher Scientific. MiR-101 mimic is a chemically modified short double-stranded RNA molecule that mimics endogenous mature miR-101 and thus artificially represses the translation of target mRNA, whereas mimic negative control #1 is a double-stranded RNA molecule that does not target any gene in human, mouse, or rat. Conversely, miR-101 inhibitors are chemically modified single-stranded oligonucleotides that bind specifically to endogenous miR-101 and artificially increase the translation of target mRNAs by inhibiting their activity. In addition, miRNA inhibitor negative control #1 is a single-stranded oligonucleotide that does not target any gene in human, mouse, or rat. Transfection of miRNA was performed using Lipofectamine RNAiMAX reagent (Thermo Fisher Scientific) according to the manufacturer’s instructions.

### Western Blotting Analysis

After rinsing with ice-cold PBS, the cells were treated with ice-cold lysis buffer (20 mM HEPES, 120 mM NaCl, 5 mM EDTA, 1% Triton X-100, 10% glycerol, 10 mM dithiothreitol, 0.5 mM phenylmethylsulfonyl fluoride, and 5 μg/ml leupeptin), and protein concentrations were determined using the Bradford assay. Equal amounts of total protein were separated using sodium dodecyl sulfate-polyacrylamide gel electrophoresis and then transferred to nitrocellulose blotting membranes. Blocking was performed at room temperature for 30 min in Tris–buffered saline with 0.05% Tween 20 containing 5% skim milk (Yukijirushi, Tokyo, Japan), followed by overnight incubation at 4°C with primary antibodies against SEL1L (1:500), HRD1 (1:1,000), or β-actin (1:2,000) in Tris–buffered saline with 0.05% Tween 20. The appropriate secondary antibodies [anti-mouse antibodies against SEL1L (1:1,000), and anti-rabbit antibodies against HRD1 and β-actin (1:5,000)] were used, and proteins were then visualized via chemiluminescence (ECL Prime, GE Healthcare, Little Chalfont, United Kingdom). Blot images were acquired using ChemiStage CC-16 (KURABO, Osaka, Japan). The intensity of protein expression, which was analyzed using ImageJ software (National Institutes of Health, Bethesda, MD, United States), was corrected by the respective β-actin expression.

### Statistical Analysis

Quantitative data were presented as the means ± standard error of the mean. Data were analyzed using Student’s *t* test or one-way analysis of variance, followed by Dunnett’s two-tailed test or Tukey–Kramer’s two-tailed test. Probability values of <0.05 were considered statistically significant. All statistical analyses were performed using IBM SPSS Statistics (version 26, IBM, Armonk, NY, United States).

## Results

### Search for MicroRNAs Targeting Suppressor/Enhancer Lin-12-Like Using Publicly Available Databases

**FIGURE 1:**
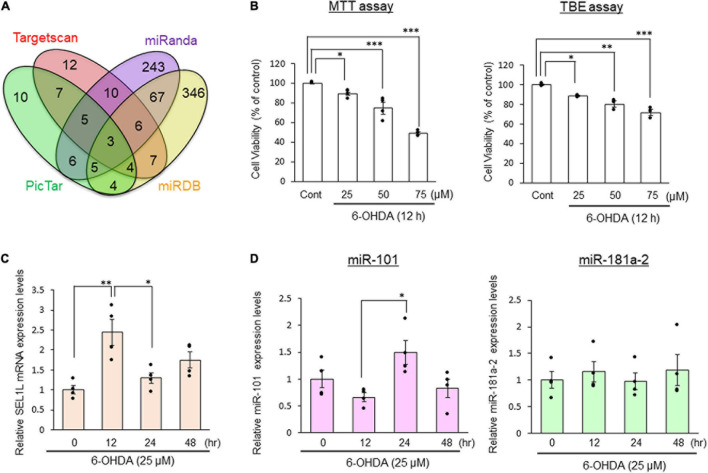
MiR-101 expression is inversely correlated with the mRNA expression of suppressor enhancer Lin12 1-like (SEL1L). **(A)** Potential targets of SEL1L predicted by the interrogation of four databases (Targetscan, PicTar, miRanda, and miRDB). **(B)** SH-SY5Y cells were stimulated with 25, 50, or 70 μM 6-hydroxydopamine (6-OHDA) for 12 h. Data on cell viability are expressed as means ± standard error of the mean of three (TBE assay) or four (MTT assay) independent experiments performed in duplicate. *p < 0.05, ^**^p < 0.01, and ^***^p < 0.001, one-way analysis of variance with Dunnett’s *post hoc* test. **(C,D)** SH-SY5Y cells were stimulated with 25 μM 6-OHDA for 12, 24, or 48 h. The relative expression levels of SEL1L mRNA, miR-101, and miR-181a-2 are presented as means ± standard error of the mean of at least four independent experiments performed in duplicate. *p < 0.05 and **p < 0.01, one-way analysis of variance with Tukey’s *post hoc* test.

We searched for candidate target miRNAs of SEL1L mRNA using information available in four databases (Targetscan, PicTar, miRanda, and miRDB; Figure 1A) and identified three miRNAs (miR-101, miR-181a-2, and miR-181b) from these databases. In addition, miR-155 and miR-183 were previously reported as miRNAs that target SEL1L (Cardano et al., 2011; Liu et al., 2014). Therefore, a total of five miRNAs were selected as candidate miRNAs of SEL1L. However, real-time PCR revealed that the cycle threshold (Ct) value exceeded 35 for miR-181b, miR-183, and miR-155 in unstimulated SH-SY5Y cells. Given that Ct is defined as the cycle number at which the fluorescence emission exceeds a fixed threshold and that a Ct value above 35 is considered to indicate a very low level of expression, we excluded miR-181b, miR-183, and miR-155 from the list of candidate miRNAs of SEL1L in subsequent analyses (Chen et al., 2009; Gallo et al., 2012).

### Evaluation of the Candidate MicroRNAs of Suppressor/Enhancer Lin-12-Like Using Quantitative Real-Time Polymerase Chain Reaction

To assess the cytotoxicity of 6-OHDA, which is commonly used to generate the cellular PD model (Latchoumycandane et al., 2011) and has been shown to induce oxidative and ER stress which result in neuronal cell death (Ryu et al., 2002; Schober, 2004), SH-SY5Y cells were treated with various concentrations of 6-OHDA (0–75 μM) for 12 h and cell viability was evaluated by the MTT and TBE assays (Figure 1B). Exposure to 6-OHDA induced cell death in a concentration-dependent manner.

We investigated whether there was a relationship between SEL1L mRNA and candidate miRNA expression levels following 6-OHDA treatment. We examined the expression of SEL1L mRNA and candidate miRNAs (miR-101 and miR-181a-2) at 0, 12, 24, and 48 h after treatment with 25 μM 6-OHDA. SEL1L mRNA expression after the 6-OHDA treatment for 12 h was significantly higher than that before the treatment (*p* < 0.01), whereas SEL1L mRNA expression was significantly lower after the 24 h treatment than the 12 h treatment (*p* < 0.05, Figure 1C). In contrast, miR-101 expression was unchanged after the 6-OHDA treatment up to 12 h, but it was significantly upregulated after the 24 h treatment (*p* < 0.05, Figure 1D). MiR-181a-2 expression was unchanged by the 6-OHDA exposure.

### miR-101 Directly Targets Suppressor/Enhancer Lin-12-Like

**FIGURE 2:**
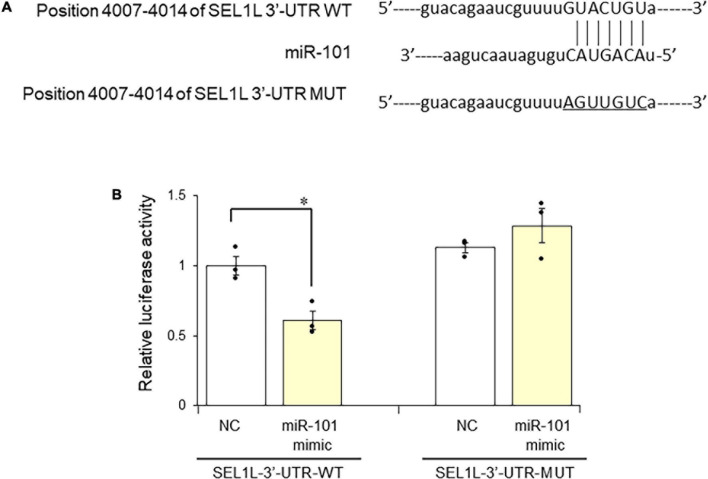
MiR-101 regulates the suppressor enhancer Lin12 1-like (SEL1L) 3′-untranslated region (3′-UTR). **(A)** Schematic diagram of the miR-101 target site in the 3′-UTR of SEL1L mRNA. **(B)** Luciferase reporter assay was performed in SH-SY5Y cells cotransfected with vectors carrying the wild-type (pmirGLO-SEL1L-3′-UTR-WT) or mutant (pmirGLO-SEL1L-3′-UTR-MUT) SEL1L 3′-UTR construct together with the miR-101 mimic or negative control (NC) for 24 h. Data are presented as means ± standard error of the mean of the relative ratio of firefly luciferase activity to *Renilla* luciferase activity in three independent experiments performed in triplicate. *p < 0.05, Student’s *t* test.

Based on our finding that miR-101 expression was inversely correlated with SEL1L expression, we next aimed to confirm that miR-101 could directly target SEL1L using a luciferase reporter assay. We cotransfected the vector containing the target region of miR-101 among the 3′-UTR of SEL1L (pmirGLO-SEL1L-3′-UTR-WT) with the mimic negative control or miR-101 mimic into SH-SY5Y cells. We found that miR-101 mimic repressed the luciferase activity of pmirGLO-SEL1L-3′-UTR-WT whereas a similar repression was not observed in the cells cotransfected with mimic negative control (*p* < 0.05). In addition, the cells cotransfected with miR-101 mimic and pmirGLO-SEL1L-3′-UTR-MUT, in which the mutation in the binding region for miR-101 prevents miR-101 binding, miR-101 mimic did not repress the luciferase activity of pmirGLO-SEL1L-3′-UTR-MUT (Figures 2A,B).

### miR-101 Negatively Regulates Suppressor/Enhancer Lin-12-Like Protein Expression and Regulates 6-OHDA–Induced Cytotoxicity in SH-SY5Y Cells

**FIGURE 3:**
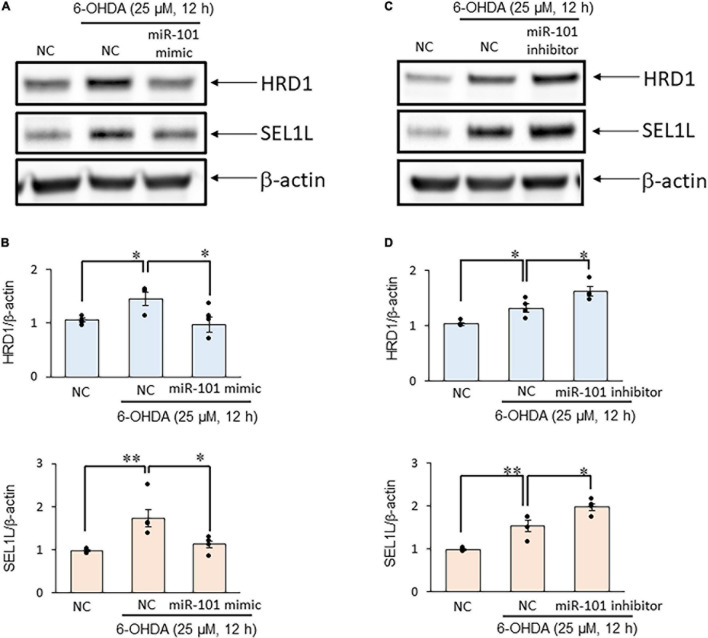
MiR-101 affects suppressor/enhancer lin-12-like (SEL1L) protein expression in a cellular model of Parkinson’s disease (PD). **(A,C)** SH-SY5Y cells were transfected with the negative control (NC), miR-101 mimic, or miR-101 inhibitor for 48 h and then stimulated with 25 μM 6-hydroxydopamine (6-OHDA) for 12 h. Representative western blots of SEL1L, HMG-CoA reductase degradation 1 (HRD1), and β-actin are shown. **(B,D)** Immunoreactive bands were quantified and expressed as means ± standard errors of the mean of four independent experiments. *p < 0.05 and ^**^p < 0.01, one-way analysis of variance with Tukey’s *post hoc* test.

**FIGURE 4:**
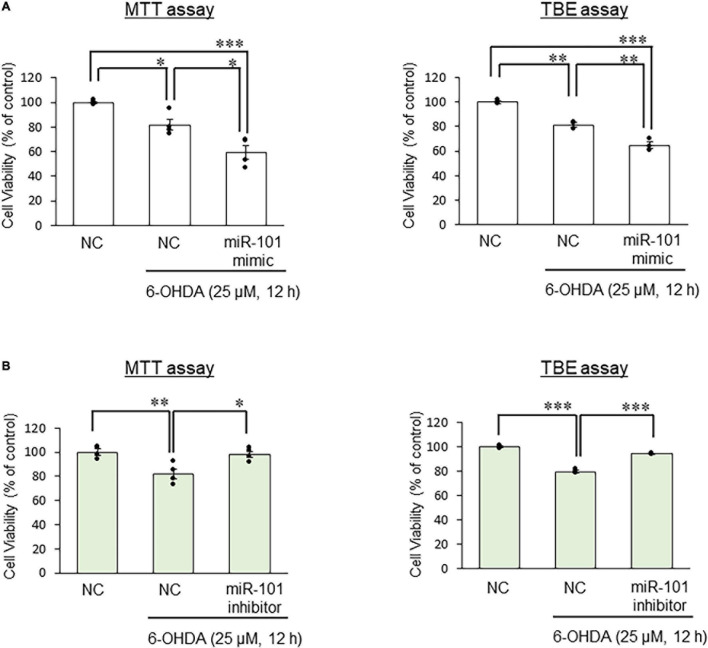
Effects of miR-101 on 6-hydroxydopamine (6-OHDA)–induced cell death. **(A,B)** SH-SY5Y cells were transfected with the negative control (NC), miR-101 mimic, or miR-101 inhibitor for 48 h and then stimulated with 25 μM 6-OHDA for 12 h. Changes in cell viability was expressed as means ± standard error of the mean of three (TBE assay) or four (MTT assay) independent experiments performed in duplicate. *p < 0.05, ^**^p < 0.01, and ^***^p < 0.001, one-way analysis of variance with Tukey’s *post hoc* test.

Finally, we examined SEL1L protein expression levels in SH-SY5Y cells to investigate the interaction between miR-101 and SEL1L protein. In initial studies using unstimulated SH-SY5Y cell extracts, we optimized experimental conditions for each antibody and tested signal linearity over a range of protein concentrations (Supplementary Figure 1). We confirmed that 6-OHDA treatment (25 μM for 12 h) significantly increased SEL1L and HRD1 protein expression levels. The overexpression of miR-101 mimic suppressed the upregulation of SEL1L and HRD1 protein expression levels induced by 6-OHDA (both *p* < 0.05, Figures 3A,B), whereas miR-101 inhibitor transfection significantly enhanced the upregulation in the expression levels of SEL1L and HRD1 (both *p* < 0.05, Figures 3C,D). We also demonstrated that miR-101 overexpression significantly increased 6-OHDA–induced cell death (*p* < 0.05 in MTT assay and *p* < 0.01 in TBE assay) and that miR-101 inhibition suppressed 6-OHDA–induced cell death (*p* < 0.05 in MTT assay and *p* < 0.001 in TBE assay, Figures 4A,B).

## Discussion

One of the possible pathogenic mechanisms involved in the PD etiology is the loss of dopaminergic neurons caused by ER stress (Mou et al., 2020). Mammalian cells activate UPR to counteract ER stress, and the activities of the UPR system include the degradation and removal of unfolded proteins via the activation of ubiquitin ligase in the ER (Hershko and Ciechanover, 1998; Yoshida, 2007; Omura et al., 2013). We have previously investigated the relationship between HRD1 (ubiquitin ligase)/SEL1L (HRD1 stabilizer) and PD and have found that 6-OHDA–induced cell death is enhanced when SEL1L expression is downregulated compared with the findings when HRD1 expression is downregulated (Omura et al., 2018). Meanwhile, associations between miRNAs and neurodegenerative diseases have recently been revealed, and several miRNAs involved in the loss of dopaminergic neurons in PD have been reported (Thome et al., 2016; McMillan et al., 2017). In this study, we searched for miRNAs that regulated SEL1L expression in a human neuronal cell line and examined whether the candidate miRNAs affect viability in a cellular model of PD. This study results suggested that miR-101 regulates SEL1L expression by targeting its 3′-UTR, indicating that miR-101 inhibition may have an inhibitory effect on neuronal cell death in PD.

In addition to miR-101, miR-181a-2, and miR-181b, which were reported to target the 3′-UTR of SEL1L mRNA in all four databases, we included miR-155 and miR-183 as candidates regulating SEL1L expression in the present study. First, miR-155 has been reported to regulate SEL1L expression in pancreatic ductal adenocarcinoma cells (Liu et al., 2014) whereas miR-183 has been shown to regulate SEL1L and to be involved in the maintenance and lineage determination of neural progenitor cells in mouse neural stem cells (Cardano et al., 2011). Therefore, these five miRNAs were selected.

Recent studies have shown that PD cellular models created using lower doses of neurotoxins, such as 1-methyl-4-phenylpyridinium ion, do not exhibit excessive cell death (Chen et al., 2015; Miyara et al., 2016). In fact, the typical symptoms of PD, such as movement disorders, are believed to occur after the loss of 50% dopaminergic neurons (mild stage), which is equal to a 70–80% decrease in striatal dopamine content in patients with PD (Dunnett and Bjorklund, 1999). However, recent studies have reported that non-motor symptoms, such as constipation, depression, olfactory disturbance, and sleep disturbance, appear prior to motor symptoms (in the early motor or premotor stage) and that these symptoms appear after the loss of about 20–30% of dopamine neurons (Schapira et al., 2017; Yilmaz et al., 2019; Ugrumov, 2020). In the present study, we used 6-OHDA at a concentration of 25 μM to model early stage PD before the appearance of motor symptoms. Identification of miRNAs that are dysregulated in early disease stage can lead to the early detection of PD, raising the possibility of developing more effective or preventive early treatment approaches. Furthermore, in a preliminary study, we confirmed that the SEL1L mRNA expression levels were higher in SH-SY5Y cells exposed to 25 μM 6-OHDA for 12 h compared to those exposed to 50 or 75 μM (Supplementary Figure 2). We previously reported that SEL1L inhibits 6-OHDA-induced cell death (Omura et al., 2018), and we thought that the increase in SEL1L might indicate resistance to 6-OHDA against cell death. Therefore, the lower concentration of 6-OHDA (25 μM) was used in the present study.

Intriguingly, the upregulation of SEL1L mRNA before the upregulation of miR-101 following treatment of SH-SY5Y cells with 6-OHDA can be explained by several mechanisms. For example, miRNAs and their target mRNAs have been demonstrated to be induced by the same transcription factors and that mature miRNAs degrade their target mRNAs (Lai et al., 2016). We have previously found that activating transcription factor 6 (ATF6), an initiation factor of UPR, is a transcription factor for the SEL1L gene (Kaneko et al., 2007). WhileATF6 might transcriptionally upregulate miR-101 together with SEL1L, the current study findings suggest that the time required for the maturation of miR-101 might be longer than that of SEL1L.

Because inserting the full-length SEL1L 3′-UTR, >4,000 bp in length, into the luciferase vector was challenging, we subcloned approximately 500 bp containing the target site. Then, we performed the luciferase assay using miR-101 mimic. MiR-101 mimic repressed the luciferase activity of SEL1L 3′-UTR, indicating that miR-101 acted directly on the SEL1L 3′-UTR target site.

Our experiments investigating the changes in protein levels of SEL1L confirmed that miR-101 regulated SEL1L protein expression and that miR-101 inhibitor had an inhibitory effect on neuronal cell death in the PD model by increasing SEL1L protein expression through the functional inhibition of endogenous miR-101.

We found that the miR-101 mimic suppressed the 6-OHDA–induced increase in HRD1 expression, and that the miR-101 inhibitor further increased this upregulation. We have confirmed the absence of miR-101 target site in the HRD1 3′-UTR in all examined databases (data not shown). We have previously reported that SEL1L knockdown via RNA interference decreases HRD1 expression and the increase of SEL1L expression results in upregulated HRD1 protein expression (Omura et al., 2012, 2018). Therefore, we speculated that the expression level of SEL1L was upregulated when the miR-101 inhibitor was transfected into SH-SH5Y cells. This phenomenon, in turn, stabilized HRD1 and increased its expression. However, when SEL1L expression was suppressed by the transfection of miR-101 mimic, HRD1 could not interact with SEL1L and became structurally unstable, resulting in the degradation of HRD1. It has also been reported that SEL1L stabilizes HRD1 and forms a complex with ERAD-related molecules such as osteosarcoma amplified 9, which recruits unfolded proteins, and bridges these molecules to HRD1 (Christianson et al., 2008). In other words, the miR-101–mediated suppression of SEL1L expression could reduce HRD1 expression and potentially also disrupt the recruitment mechanism of denatured proteins. However, this concept must be clarified in future research.

Although the expression profile of miRNA differs depending on the tissue (Guo et al., 2014), miR-101 is abundantly expressed in the brain (Llorens et al., 2013). In addition, it has been recently reported that LncRNA-T199678, a long ncRNA, mitigates α-synuclein–induced dopaminergic neuron injury by downregulating miR-101 (Bu et al., 2020). PD is accompanied by excessive inflammation, and the ncRNA myocardial infarction-associated transcript 2, which relieves inflammation, suppresses the accumulation of miR-101 induced by tumor necrosis factor alpha and alleviates apoptosis (Han et al., 2019). The results of our study are consistent with those of these previous reports. Therefore, miR-101 may play an important role in the pathogenesis and progression of PD through the regulation of SEL1L expression. In other words, miR-101 could be used as a biomarker of PD. Future studies are needed to clarify the alteration of miR-101 levels in the blood of patients with PD.

The present study has some limitations. Given that this study only examined SH-SY5Y cells, further studies are needed to determine whether similar results can be obtained with other neuronal cell lines. Also, studies should examine whether miR-101 or SEL1L levels change with the use of 1-methyl-4-phenylpyridinium, which has also been used to generate a cellular model of PD, or whether miR-101 and SEL1L levels change in a mouse model of PD.

In conclusion, this study found that miR-101 regulates SEL1L expression in a cellular model of PD. Because SEL1L is a key molecule in neurodegenerative disorders such as PD, miR-101 might represent a therapeutic target for the treatment of PD and other neurodegenerative disorders related to ER stress.

## Data Availability Statement

Publicly available datasets were analyzed in this study. This data can be found here: http://mirdb.org/, https://pictar.mdc-berlin.de/, http://www.targetscan.org/vert_71/, and https://bioweb.pasteur.fr/packages/pack@miRanda@3.3a.

## Author Contributions

TO, LN, RW, and HN performed the experiments. TO, HN, KY, SI, and SN contributed to the analysis and interpretation. TO, LN, KY, SI, KI, SN, AY, TN, JK, IY, and KM drafted the work. All authors approved the final version of the manuscript.

## Conflict of Interest

The authors declare that the research was conducted in the absence of any commercial or financial relationships that could be construed as a potential conflict of interest.

## Publisher’s Note

All claims expressed in this article are solely those of the authors and do not necessarily represent those of their affiliated organizations, or those of the publisher, the editors and the reviewers. Any product that may be evaluated in this article, or claim that may be made by its manufacturer, is not guaranteed or endorsed by the publisher.

## Abbreviations

PD: Parkinson’s disease
microRNA: miRNA
ER: endoplasmic reticulum
UPR: unfolded protein response
ERAD: ER-associated degradation
miRNA: microRNA
HRD1: HMG-CoA reductase degradation 1
SEL1L: suppressor enhancer Lin12 1-like
Pael-R: Parkin-associated endothelin receptor-like receptor
6-OHDA: 6-hydroxydopamine
ATF6: activating transcription factor 6.
