# Supplementary material for: MicroRNA-101 Regulates 6-Hydroxydopamine-Induced Cell Death by Targeting Suppressor/Enhancer Lin-12-Like in SH-SY5Y Cells
Source: Front Mol Neurosci. 2021 Dec 9;14:748026. doi: 10.3389/fnmol.2021.748026 (PMC8695805; doi:10.3389/fnmol.2021.748026)
Supplement: Supplementary file 1 [file Image_1.pdf]

Supplementary Material

MicroRNA-101 regulates 6-hydroxydopamine-induced cell death by targeting SEL1L in SH-SY5Y cells

This docx. File includes:

Figs. S1 to S2

Figure S1

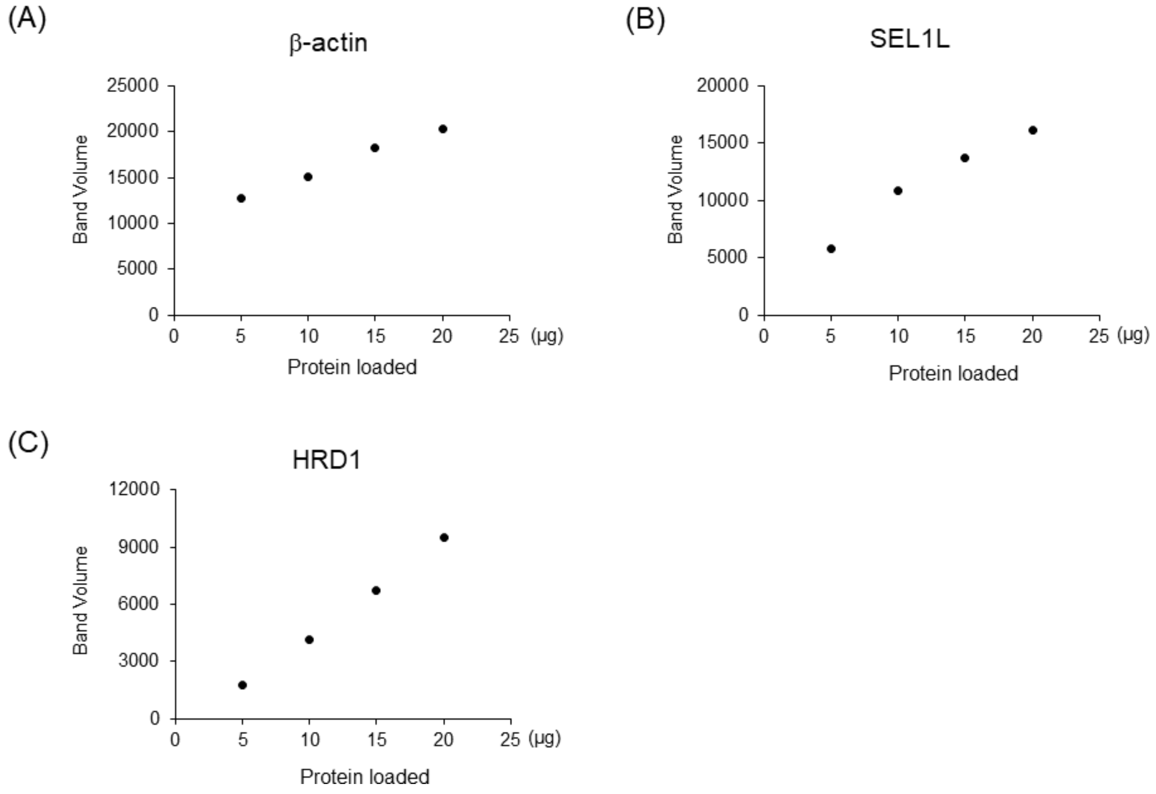

Figure S1. Confirmation of the linearity of western blot signals for all antibodies used in this study. (A–C) Whole-cell lysates of unstimulated SH-SY5Y cells were analyzed by western blotting. Signal intensity of each protein amount (5, 10, 15, or 20 μg) for each antibody were measured by ImageJ software (A, anti-β-actin; B, anti-SEL1L antibody; C, anti-HRD1 antibody).

Figure S2

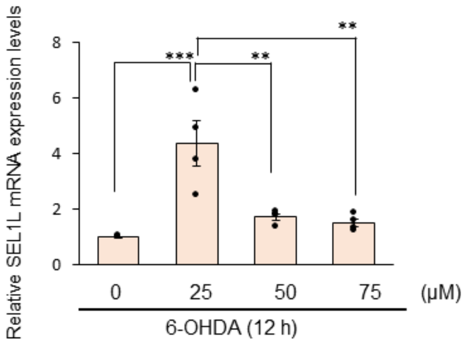
Relative SEL1L mRNA expression levels
8
6
4
2
0
***
**
**
0
25
50
75
(μM)
6-OHDA (12 h)
